# Association between mouth-breathing and atypical swallowing in young orthodontic patients at Egas Moniz Dental Clinic

**DOI:** 10.1080/07853890.2021.1897421

**Published:** 2021-09-28

**Authors:** A. R. Barata, G. Kizi, V. Alves, L. Proença, A. Delgado

**Affiliations:** a Centro de Investigação Interdisciplinar Egas Moniz (CiiEM), Egas Moniz Cooperativa de Ensino Superior, Caparica, Portugal; b Consulta Assistencial de Ortodontia, Egas Moniz Dental Clinic (EMDC), Egas Moniz Cooperativa de Ensino Superior, Caparica, Portugal

## Abstract

**Introduction:**

Breathing and swallowing are functions of the stomatognathic system, since they are vital and innate to the human being, moreover, interrelated [1,2]. Most of the breathing is nasal, however mouth-breathing can occur, being a pathological adaptation that can lead to a series of changes, often irreversible, in the growth and development of a child, for instance causing changes in swallowing [3]. This mechanism differs between children and adults and, if identified in a child, up to five years, it is considered as infantile deglutition pattern being replaced by mature deglutition pattern after this period [4]. If it continues beyond the period in which it is considered normal, it is renamed as atypical [5]. Therefore, the aim of this study was to explore the possible relation between mouth-breathing and atypical swallowing in children.

**Materials and methods:**

The study took place between January 2018 to February 2019, involving patients referred to the Care Consultation of Orthodontics Egas Moniz Dental Clinic (EMDC). The sample consisted of 86 patients, that *n* = 46 (53.4%) were females and *n* = 40 (46.6%) were males, with a mean age of 12.3 years. The study was approved by an Ethics Committee of Egas Moniz. Inclusion criteria were: children of both sexes aged 5–18 years, having clinical record at EMDC and with the correspondent informed consent signed by the parents. Exclusion criteria were: children who had past orthodontic treatment and having craniofacial anomalies or syndromes. The method used to assess breathing and swallowing patterns was adapted by Marchesan [2]. Data were analysed by using descriptive and inferential methodologies (chi-square test). A significance level of 5% was established in the latter case.

**Results:**

A higher prevalence of mouth-breathers was found in women *n* = 49 (56.5%) than in men *n* = 37 (43.5%). Atypical swallowing, was also more prevalent in women *n* = 48 (56.1%) than in men *n* = 38 (43.9%), however, in both cases, the differences were not found to be statistically significant (*p* = .594, *p* = .570). *n* = 52 (83.9%) of the mouth-breathers were identified as having atypical swallowing, conversely to *n* = 14 (58.3%) of the normal (nasal) breathers. Overall, there was a statistically significant association between mouth-breathing and patients with atypical swallowing (*p* = .012).

**Discussion and conclusions:**

The results show that mouth-breathing and atypical swallowing were found to be significantly associated *n* = 52 (83.9%) in this study, being closed to Lemos et al. who found higher results (97.2%) [1]. Other studies reported that is not statistically significant the association between mouth-breathing and atypical swallowing [6]. The results of this study are clinically relevant and encouraging the search for early diagnosis and intervention.
